# (Poly)phenolic Content and Profile and Antioxidant Capacity of Whole-Grain Cookies are Better Estimated by Simulated Digestion than Chemical Extraction

**DOI:** 10.3390/molecules25122792

**Published:** 2020-06-17

**Authors:** Francesca Danesi, Luca Calani, Veronica Valli, Letizia Bresciani, Daniele Del Rio, Alessandra Bordoni

**Affiliations:** 1 Department of Agricultural and Food Sciences (DISTAL), University of Bologna, Piazza Goidanich 60, 47521 Cesena, Italy; francesca.danesi@unibo.it (F.D.); valli.veron@gmail.com (V.V.); 2 Interdepartmental Center for Agri-food Industrial Research (CIRI Agrifood), University of Bologna, Via Quinto Bucci 336, 47521 Cesena, Italy; 3 Department of Food and Drug, University of Parma, Parco Area delle Scienze 27/A, 43124 Parma, Italy; luca.calani@unipr.it; 4 Department of Veterinary Science, University of Parma, Medical School, Building C, Via Volturno 39, 43125 Parma, Italy; letizia.bresciani@unipr.it (L.B.); daniele.delrio@unipr.it (D.D.R.)

**Keywords:** whole-grain wheat, bioaccessibility, antioxidants, (poly)phenols, durum wheat, KAMUT^®^ khorasan wheat, simulated digestion, sourdough fermentation

## Abstract

It is widely recognized that the biological effects of phytochemicals cannot be attributed to the native compounds present in foods but rather to their metabolites endogenously released after intake. Bioavailability depends on bioaccessibility, which is the amount of the food constituent that is released from the matrix in the gastrointestinal tract. The use of chemical extraction to evaluate the content and profile of phytochemicals does not mirror the physiological situation in vivo, and their bioaccessibility should be considered while assessing their nutritional significance in human health. The current study was designed to compare the (poly)phenolic profile and content and antioxidant capacity of whole-grain (WG) cookies using chemical extraction and a more physiological approach based on simulated digestion. Three types of organic WG cookies (made with durum, Italian khorasan, or KAMUT^®^ khorasan wheat) were considered, either fermented by *Saccharomyces Cerevisiae* or sourdough. Although the flour type and the fermentation process influenced the release of phytochemicals from the cookie matrix, in almost all samples, the simulated digestion appeared the most efficient procedure. Our results indicate that the use of chemical extraction for evaluation of the phytochemicals content and antioxidant capacity of food could lead to underestimation and underline the need for more physiological extraction methods.

## 1. Introduction

Phytochemicals are bioactive non-nutrient components of fruits, vegetables, grains, and other plant foods that may have biological significance and provide desirable health benefits beyond basic nutrition, reducing the risk of major chronic diseases [1]. (Poly)phenolic compounds (PCs) represent one of the major groups of phytochemicals.

It is widely recognized that most of the biological effects of PCs cannot be attributed to the native compounds present in foods but rather to their metabolites of various origins gaining contact with the internal compartments after food intake [2]. Therefore, the health benefits of dietary PCs depend not only on their concentration in the food but mainly on their absorption, metabolism, and availability in the target tissue [3]. Bioavailability, i.e., the proportion of a food constituent that is absorbed and utilized in the normal metabolism [4], depends on bioaccessibility, which is the amount of the food constituent that is released from the solid food matrix in the gastrointestinal tract [5]. The bioaccessibility of PCs is greatly influenced by their interactions with the food matrix. In turn, these interactions depend on the physicochemical properties of the food matrix (e.g., pH, temperature, texture), on the presence of other components that can improve or antagonize accessibility [6], and on the technologies used for food processing [7,8].

The majority of studies examining the PC content or the antioxidant activity of foods do not consider bioaccessibility and rely on non-physiological extraction conditions [9] that often ignore PCs remaining in the residues of the chemically extracted material. Recent studies evidenced that these non-extractable bioactive molecules represent a major part of total dietary PCs, and exhibit a substantial biological activity [10]. Therefore, the concentration and profile of PCs in food after chemical extraction do not mirror the physiological situation in vivo [11], and could give misleading results while assessing their nutritional significance in human health.

In the current study, we compared two different approaches to evaluate the PC profile and antioxidant capacity of organic whole-grain (WG) cookies: a standard approach based on chemical extraction and a more physiological approach based on in vitro-simulated digestion. Simulated digestion was followed by ultrafiltration, allowing the selection of compounds that were small enough to be potentially absorbable through the intestinal mucosa (<3 kDa digested fraction, as bioaccessible fraction) [12]. WGs are concentrated sources of phytochemicals, mainly PCs [13], and cookies were chosen as the model food since they are a popular foodstuff, consumed by a wide range of populations [14].

Three different types of organic WG cookies were considered: i) WG durum wheat cookies (DUR); ii) WG Italian khorasan wheat cookies (KHO); and iii) WG KAMUT^®^ khorasan wheat cookies (KAM). To be sold under the trademark KAMUT^®^, khorasan wheat must meet specific standards that so far are consistently satisfied only in the growing region of North America [15,16]. In this study, we used Italian khorasan wheat and KAMUT^®^ khorasan wheat to evaluate the impact of environmental factors on the content and bioaccessibility of PCs.

**Figure 1:**
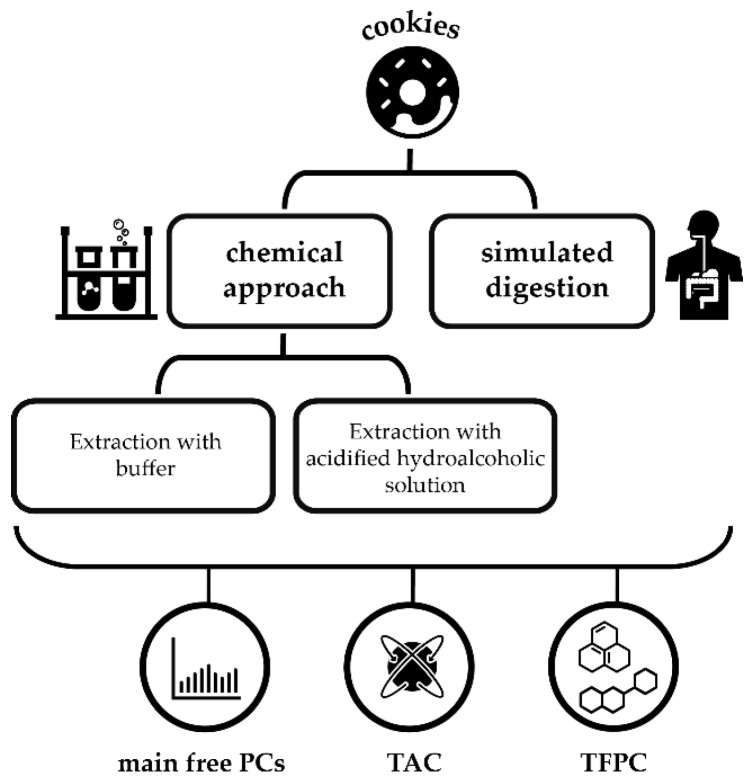
Schematic representation of the approaches used to estimate the (poly)phenolic profile (as main free PCs), the antioxidant capacity (TAC), and the total free PC content (TFPC) of the WG cookies.

To verify the possible influence of production processes [17], the WG cookies were prepared by standard fermentation using *Saccharomyces cerevisiae* (DUR_S_, KHO_S_, KAM_S_) or sourdough fermentation using *S. cerevisiae* and lactic acid bacteria (LAB) (DUR_L_, KHO_L_, KAM_L_). The study design is schematically reported in Figure 1.

## 2. Results and Discussion

The ultra-high-performance liquid chromatography-mass spectrometry (uHPLC-MS^n^) allowed the identification of seventeen PCs among free (poly)phenol fraction of the investigated cookies (Table 1). In detail, six compounds were phenolic acids, mainly hydroxycinnamates, while the remaining compounds were flavones, mainly apigenin-*C*-diglycosides. Among flavones, five compounds were quantified as the most abundant flavonoids in the investigated samples.

Since hydrolysable phenolic acids are very abundant in WG flours and are generally predominant in the bound form [18], we also evaluated the composition and abundance of bound forms of PCs through a preliminary chromatographic analysis of the experimental cookies treated with alkaline hydrolysis (Supplementary Materials, Table S1). Regardless of the type of WG cookie, ferulic and diferulic acid were the major bound PCs, as previously observed in *Triticum* species [19,20]. The identification of diferulates was performed based on mass spectral characteristics previously reported [21]. Bound ferulic and diferulic acid present in KAM cookies were approximately 2/3 of those present in DUR and KHO cookies, while no apigenin-*C*-diglycosides were recovered in the bound (poly)phenol fraction. Differently, apigenin-*C*-hexoside-*C*-pentoside isomers were the dominant PCs in the free forms, mainly in DUR cookies, showing that the WG cookies prepared with Italian durum wheat had the highest content of PC free forms, which can be enhanced after LAB fermentation. The identification of several apigenin-*C*-diglycosides among the free (poly)phenol fraction confirmed the previous findings on wheat-derived food products [22,23,24,25,26]. Two isomers of apigenin-*C*-hexoside-*C*-pentoside were the most abundant apigenin-*C*-diglycosides, which could be schaftoside and isoschaftoside, the most relevant wheat-derived flavonoids [22,23,24,25,26]. Sinapic acid was detected in the fraction of bound PCs and was absent in the free form. *p*-Coumaric acid barely existed in the free form as previously reported by other authors [27] and it was detected only in KHO_L_ cookies. Ferulic acid was released from their bound form by sourdough fermentation only in WG cookies made with DUR and KHO, where the highest content of this phenolic acid was observed. A higher release of phenolic acids after sourdough than standard fermentation of WG cereals was previously reported in barley [28], wheat and rye malt [29], and in bread made with wheat, spelt, or rye flours [30], and it was ascribed to LAB feruloyl esterase activity [30,31]. Our results evidenced that the effect of sourdough fermentation was modulated by the cereal type.

The influence of the chemical extractions or the simulated digestion on the PC profile of the WG cookies prepared using standard or sourdough fermentation are reported in Table 2 and Table 3, respectively. These data clearly show that the simulated digestion was not only the most physiological but also the most exhaustive approach to release single PCs, mainly *p*-coumaric and ferulic acid, from the food matrix. The higher concentration of free PCs quantified in digested cookies than hydroalcoholic extracts is consistent with a previous study on different types of bread [31]. Similarly, the treatment of cereal-derived products with digestive enzymes, including pepsin, pancreatin, amylase, and amyloglucosidase, led to a significant increase of the total phenolic content compared to the aqueous-organic extracts [32].

**Figure 2:**
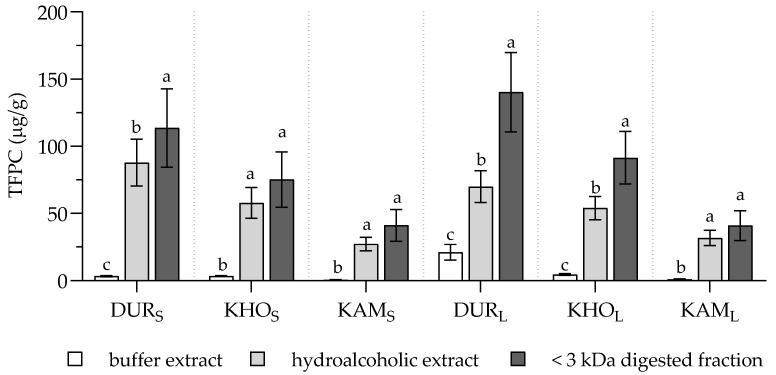
Total free PC content (TFPC) of buffer and hydroalcoholic extracts and <3 kDa digested fractions of the experimental cookies. TFPC represents the sum of total free PCs determined by uHPLC-MS^n^ and is expressed as µg/g of cookie. Values are expressed as mean ± SD (*n* = 4 for extracts, *n* = 3 for digested samples). Statistical analysis was by two-way ANOVA followed by Tukey’s HSD test. Within each cookie type, different letters indicate statistical significance among samples (*p* < 0.05).

Even considering the total free PC content (TFPC) of samples (i.e., the sum of total free PCs determined by uHPLC-MS^n^), in all cookies’ extraction, buffer was not efficient, confirming that the best solvent for chemical extraction of complex food should include a hydrophilic and a lipophilic phase (Figure 2). Hydroalcoholic extraction and simulated digestion were equally efficient in samples with the lowest TFPC (KHO_S_, KAM_S_, and KAM_L_), while with the increase of TFPC the simulated digestion was the most exhaustive process even in this case. An effect of both variables was detected (*F* (5, 48) = 32.41 and *F* (2, 48) = 206.8 for types of WG cookie and type of extraction, respectively), with interaction between the two variables (*F* (10, 48) = 7.661).

Of note, in the herein reported study, we showed that simulated gastrointestinal digestion facilitated the release of all PCs from the food matrix more than the chemical extraction methods employed in most studies. Accordingly, further PC data analysis was mainly focused on the <3 kDa digested fractions of the experimental cookies (Supplementary Materials, Table S2). The concentration of the most represented free PCs, apigenin-*C*-hexoside-*C*-pentosides and apigenin-*C*-dihexoside (1st), was in the order DUR > KHO > KAM. The content of ferulic and *p*-coumaric acid in DUR and KHO was higher after LAB fermentation than after standard fermentation, while no differences due to fermentation were observed in KAM. Although in the <3 kDa digested fractions differences in the PC profile appeared strongly dependent on the type of flours, they were modulated by the fermentation (see Supplementary Materials, Table S3 for a complete description of the two-way analysis of variance). A significant effect of both variables (WG cereal and fermentation type) with interaction between the two variables was detected for apigenin-*C*-hexoside-*C*-pentoside (2nd), and ferulic and *p*-coumaric acid.

**Figure 3:**
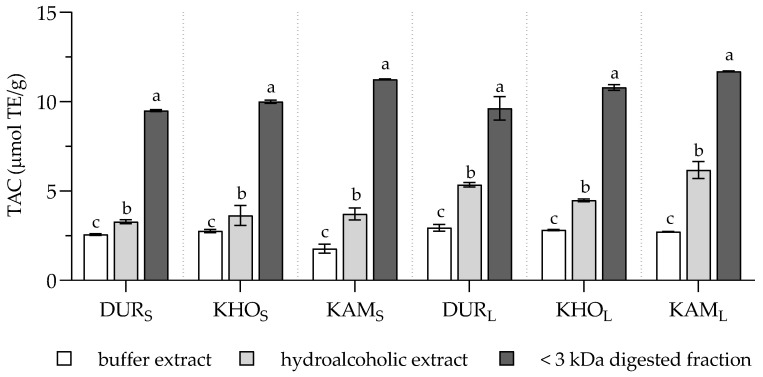
Total antioxidant capacity (TAC) of buffer and hydroalcoholic extracts and <3 kDa digested fractions of the experimental cookies. Values are expressed as mean ± SD (*n* = 3). Statistical analysis was by two-way ANOVA followed by Tukey’s HSD test. Within each cookie type, different letters indicate statistical significance among samples (*p* < 0.05).

Since the antioxidant properties of foods may depend on their release from the food matrix during the digestion process and may greatly differ from those obtained by the chemical extractions, the total antioxidant capacity (TAC) of the chemical extracts and digested fraction was assessed. Regardless of the type of cookie, TAC was about two-fold higher in digested samples than in the corresponding chemical extracts (Figure 3), confirming the greater release of antioxidant compounds during simulated digestion than after chemical extraction previously shown in other food matrices [33,34,35]. Of note, the increased TAC in the digested samples could also be accounted for by the addition of bile during the digestion procedure since bile pigments are potent peroxyl radical scavengers [36].

As for TFPC, a significant effect of both independent variables, i.e., types of WG cookie and type of extraction, was evidenced by the two-way ANOVA (*F* (5, 18) = 31.61 and *F* (2, 18) = 2923, respectively), with interaction between the two variables (*F* (10, 18) = 16.74).

## 3. Materials and Methods

### 3.1. Materials

All chemicals, reagents, and solvents were purchased from Sigma-Aldrich Co. (St. Louis, MO, U.S.A.) unless otherwise stated. All aqueous solutions were prepared using ultrapure water (Milli-Q; Millipore, Bedford, CT, U.S.A.). All phenolic standards were purchased from Sigma-Aldrich Co. except vitexin, which was purchased from Extrasynthese (Genay Cedex, France).

### 3.2. Cookie Preparation

Six different types of WG cookies were tested: i. Italian durum wheat, standard fermentation (*S. cerevisiae*) (DUR_S_); ii. Italian khorasan wheat, standard fermentation (KHO_S_); iii. KAMUT^®^ khorasan wheat, standard fermentation (KAM_S_); iv. Italian durum wheat, sourdough fermentation (*S. cerevisiae* and LAB) (DUR_L_); v. Italian khorasan wheat, sourdough fermentation (KHO_L_); and vi. KAMUT^®^ khorasan wheat, sourdough fermentation (KAM_L_). Flours, *S. cerevisiae* and LAB strains, other cookie ingredients, and the cooking procedure are reported in Valli et al. [12].

### 3.3. Cookie Chemical Extraction

Since a single chemical procedure could not accurately reflect all the antioxidants in a complex system [37], we firstly used two different methods of chemical extraction as described in Danesi et al. [38] with some modifications. Briefly, 5 g of each sample were extracted with 20 mL ethanol/water (70:30 *v*/*v*) acidified with 0.1% HCl or with the buffer solution used for the simulated digestion procedure. After a 20 min incubation at 40 °C, samples were centrifuged at 3000× *g* for 5 min and the supernatant collected. The extraction phase was repeated with 10 mL of acidified ethanol/water or buffer, and supernatants were combined. Prior to uHPLC-MS^n^ analysis, hydro-alcoholic extracts were dried in a speed vacuum and dissolved in a mixture of methanol/acidified water (1% formic acid) (50:50, *v*/*v*). Buffer extracts were further extracted with ethyl acetate by vigorous vortex for 1 min, dried in a speed vacuum, and resuspended in methanol/acidified water (1% formic acid) (50:50, *v*/*v*). Moreover, since cereal-based products are characterized by an important fraction of bound PCs [20], an alkaline hydrolysis was also applied [21]. Each cookie sample was extracted at least in triplicate using the different procedures.

### 3.4. Cookie Simulated Digestion

WG cookies were in vitro digested, according to Bordoni et al. [39]. The digested mixture was centrifuged at 4000× *g* for 5 min, the upper lipid layer was removed, and the lower aqueous phase centrifuged again at 21,000× *g* for 20 min. The supernatant was filtered with 0.2 µm membranes, and an aliquot was sequentially ultrafiltered over a 3 kDa molecular weight cut-off semi-permeable membrane (Amicon Ultra, EMD Millipore, Billerica, MA, U.S.A.) to obtain the bioaccessible fraction (<3 kDa digested fraction).

The <3 kDa digested samples were diluted 1:1 with methanol/acidified water (1% formic acid) and mixed by vortex before uHPLC-MS^n^ sample preparation and analysis. Each cookie sample was digested in triplicate.

### 3.5. Total Antioxidant Capacity (TAC)

TAC was measured in the chemical extracts and in the <3 kDa digested fraction, as reported in Valli et al. [40]. Values obtained were expressed as µmol of Trolox equivalents (TE)/g of cookie.

### 3.6. (Poly)phenolic Profile and Content

The chemical extracts and the <3 kDa digested fractions were analyzed using an Accela uHPLC 1250 equipped with a linear ion-trap-mass spectrometer (LTQ XL, Thermo Fisher Scientific Inc., San José, CA, USA) fitted with a heated-electrospray ionization probe (H-ESI-II; Thermo Fisher Scientific Inc., San José, CA, USA). The separation was carried out by means of a C18 BlueOrchid column (50 × 2 mm; 1.8 μm particle size; Knauer, Berlin, Germany).

For uHPLC separation, mobile phase A was acidified water (0.1% formic acid) and mobile phase B was methanol/water (98/2, *v*/*v*). The gradient started with 93% A and 7% B. After 1 min at isocratic conditions, the mobile phase reached 50% of eluent A in a 12 min linear gradient. Then, phase A turned up to 80% and was maintained for 3 min, followed by 5 min of re-equilibration time at the starting conditions. The flow rate was 0.2 mL/min, the injection volume was 5 μL, and the column temperature was set at 40 °C. Initially, a preliminary investigation of the main PCs was carried out using full-scan data-dependent MS^3^ mode, scanning in a mass to charge (*m*/*z*) range of 100–1000, using collision-induced dissociation (CID) equal to 35 (arbitrary units) to obtain fragmentation. After this first step, further specific MS^2^ analyses were carried out to unambiguously identify and quantify the compounds revealed in the first step, by monitoring specific *m*/*z* transitions. Molecules were fragmented using pure helium (99.99%), with a CID setting of 15 for the production of the molecular ion, and 35 for subsequent fragmentations. Identification was performed by comparison with the literature [21]. The analyses were carried out in negative ionization mode, with a capillary temperature of 275 °C, while the source was set at 45 °C. The sheath gas flow was 40 units, while auxiliary and sweep gases were set to 5 units. The source voltage was 4 kV. The capillary voltage and tube lens were −21 and −58 V, respectively. The quantification of each PC was performed through a calibration curve built with the respective commercial standard, when available, while apigenin-*C*-glycosides were quantified as apigenin-8-*C*-glucoside (vitexin) equivalents, the most structurally related compound commercially available. The calibration curve of ferulic acid was used for diferulic acid quantification as previously reported [21].

### 3.7. Statistical Analysis

Differences among samples were tested by one-way analysis of variance (ANOVA) followed by Tukey′s HSD test or by two-way ANOVA. Statistical significance was set at *p* < 0.05.

## 4. Conclusions

Antioxidant activity, PC content, and the profile of food are usually measured after their chemical extraction from the food matrix. Our results indicate that in vitro digestion, besides better simulating the physiological condition, can be a more exhaustive procedure at least in WG cookies. This should be carefully considered while evaluating food as a source of PCs and antioxidant compounds [41].

Our findings confirmed that the flour type and the fermentation process used to bake cookies affect not only the concentration and profile [42] but also the bioaccessibility of bioactive compounds.

Research on food based on WG cereals requires a more complete understanding of the bioavailability of bioactive compounds. The comparison between commonly used chemical extraction and a physiologically appropriate in vitro model answers the need for increased knowledge in the interaction among matrix and bioactive compound bioaccessibility of WG foods.

## Figures and Tables

**Table 1:** Chromatographic and mass spectral characteristics of the free PCs identified in the experimental cookies.

Compound	RT	[M − H]^−^ (*m*/*z*)	MS^2^ ions (*m*/*z*)
***Phenolic Acids***			
***p*-Hydroxybenzoic acid**	**5.8**	**137**	**93**
***p*-Coumaric acid**	**9.5**	**163**	**119**
**Caffeic acid**	**7.7**	**179**	**135**
**Ferulic acid**	**10.3**	**193**	**149, 178, 134**
**Sinapic acid**	**10.6**	**223**	**208, 179, 164**
**Sinapic acid isomer**	**10.8**	**223**	**208, 164, 179**
***Flavones***			
Apigenin-*C*-hexoside-*C*-pentoside	9.9	563	473, 443, 503, 545, 383, 353
Apigenin-*C*-hexoside-*C*-pentoside	10.4	563	473, 443, 503, 545, 383, 353
**Apigenin-*C*-hexoside-*C*-pentoside (1st)**	**10.6**	**563**	**473, 443, 503, 545, 383, 353**
**Apigenin-*C*-hexoside-*C*-pentoside (2nd)**	**11.1**	**563**	**473, 443, 503, 545, 383, 353**
Apigenin-*C*-hexoside-*C*-pentoside	12.1	563	473, 443, 503, 545, 383, 353
Apigenin-*C*-hexoside-*C*-pentoside	12.4	563	473, 443, 503, 545, 383, 353
**Apigenin-*C*-hexoside-*C*-hexoside (1st)**	**9.7**	**593**	**473, 503, 353, 575, 383**
**Apigenin-*C*-hexoside-*C*-hexoside (2nd)**	**10.1**	**593**	**473, 503, 353, 575, 383, 533**
Apigenin-*C*-hexoside-*O*-hexoside	10.8	593	431, 473, 311, 503, 341, 413, 383
**Apigenin-*C*-hexoside-*C*-hexoside (3rd)**	**11.6**	**593**	**473, 503, 383, 575, 413, 533**
Luteolin-*O*-rutinoside	12.6	593	285; MS^3^(285): 241, 175, 199, 217, 243, 197

RT: retention time. *m*/*z*: mass-to-charge ratio. The quantified PCs are reported in bold.

**Table 2:** Concentration of the main free PCs in the experimental cookies prepared using standard fermentation. Values are expressed as mean values ± SD (*n* = 4 for extracts, *n* = 3 for digested samples). The statistical analysis was performed using one-way ANOVA followed by Tukey’s honest significant difference (HSD) test. For each cookie type, different letters in the same row indicate statistical significance (*p* < 0.05).

Compound	DUR_S_ (µg/g of Cookie)			KHO_S_ (µg/g of Cookie)			KAM_S_ (µg/g of Cookie)		
	Buffer Extract	Hydroalcoholic Extract	<3 kDa Digested Fraction	Buffer Extract	Hydroalcoholic Extract	<3 kDa Digested Fraction	Buffer Extract	Hydroalcoholic Extract	<3 kDa Digested Fraction
Apigenin-C-hex-C-pen (1st)	0.28 ± 0.07^c^	23.14 ± 1.67^b^	30.43 ± 2.29^a^	0.17 ± 0.02^c^	13.68 ± 0.22^b^	17.73 ± 0.66^a^	0.05 ± 0.06^c^	6.71 ± 0.19^b^	10.70 ± 0.36^a^
Apigenin-C-hex-C-pen (2nd)	0.80 ± 0.09^c^	54.19 ± 3.02^b^	73.87 ± 0.50^a^	0.61 ± 0.09^c^	36.09 ± 0.68^b^	49.84 ± 0.84^a^	0.19 ± 0.06^c^	15.78 ± 0.24^b^	26.65 ± 0.15^a^
Apigenin-C-dihex (1st)	nd	0.55 ± 0.05^b^	2.02 ± 0.15^a^	nd	0.56 ± 0.02^b^	1.25 ± 0.02^a^	nd	0.20 ± 0.01	nd
Apigenin-C-dihex (2nd)	nd	1.84 ± 0.10^b^	2.38 ± 0.02^a^	nd	0.79 ± 0.04	nd	nd	0.32 ± 0.00	nd
Apigenin-C-dihex (3rd)	nd	1.91 ± 0.02	nd	nd	0.37 ± 0.02	nd	nd	0.16 ± 0.01	nd
p-Hydroxybenzoic acid	0.40 ± 0.08^a^	0.25 ± 0.02^b^	nd	0.59 ± 0.09^a^	0.32 ± 0.03^b^	nd	0.27 ± 0.05^a^	0.18 ± 0.03^b^	nd
p-Coumaric acid	0.52 ± 0.03^b^	0.45 ± 0.02^b^	0.64 ± 0.06^a^	0.96 ± 0.10^a,b^	0.78 ± 0.03^b^	1.07 ± 0.15^a^	0.17 ± 0.03^c^	0.22 ± 0.00^b^	0.41 ± 0.02^a^
Caffeic acid	nd	0.13 ± 0.01	nd	nd	0.11 ± 0.00	nd	0.05 ± 0.02^a^	0.05 ± 0.00^a^	nd
Ferulic acid	1.38 ± 0.11^b^	4.72 ± 0.17^a,b^	4.24 ± 0.79^a^	1.14 ± 0.19^b^	4.80 ± 0.08^a,b^	5.30 ± 1.21^a^	nd	3.11 ± 0.05^a^	3.40 ± 0.35^a^
Sinapic acid isomers	nd	0.65 ± 0.04	nd	nd	0.38 ± 0.04	nd	nd	0.52 ± 0.07	nd

nd: not detected; hex: hexoside; pen: pentoside; dihex: dihexoside.

**Table 3:** Concentration of the main free PCs in the experimental cookies prepared using sourdough fermentation. Values are expressed as mean values ± SD (n = 4 for the chemical extracts, n = 3 for the digested samples). The statistical analysis was performed using one-way ANOVA followed by Tukey’s HSD test. For each cookie type, different letters in the same row indicate statistical significance (*p* < 0.05).

Compound	DUR_L_ (µg/g of Cookie)			KHO_L_ (µg/g of Cookie)			KAM_L_ (µg/g of Cookie)		
	Buffer Extract	Hydroalcoholic Extract	<3 kDa Digested Fraction	Buffer Extract	Hydroalcoholic Extract	<3 kDa Digested Fraction	Buffer Extract	Hydroalcoholic Extract	<3 Kda Digested Fraction
Apigenin-C-hex-C-pen (1st)	1.31 ± 1.18^c^	15.71 ± 0.32^b^	31.47 ± 1.32^a^	0.17 ± 0.04^c^	9.54 ± 0.45^b^	17.21 ± 0.23^a^	nd	7.73 ± 0.46^b^	10.77 ± 0.29^a^
Apigenin-C-hex-C-pen (2nd)	3.73 ± 3.49^c^	36.26 ± 1.02^b^	81.38 ± 0.20^a^	0.56 ± 0.09^c^	26.57 ± 0.33^b^	49.37 ± 1.15^a^	0.20 ± 0.02^c^	18.22 ± 1.00^b^	25.86 ± 0.14^a^
Apigenin-C-dihex (1st)	nd	0.34 ± 0.02^b^	2.06 ± 0.17^a^	nd	0.38 ± 0.03^b^	1.05 ± 0.07^a^	nd	0.23 ± 0.01	nd
Apigenin-C-dihex (2nd)	nd	1.09 ± 0.03	nd	nd	0.55 ± 0.01	nd	nd	0.38 ± 0.03	nd
Apigenin-C-dihex (3rd)	nd	1.23 ± 0.02^b^	2.35 ± 0.24^a^	nd	0.27 ± 0.03	nd	nd	0.18 ± 0.02	nd
p-Hydroxybenzoic acid	0.76 ± 0.12^a^	0.41 ± 0.01^b^	nd	1.36 ± 0.14^a^	0.66 ± 0.01^b^	nd	0.79 ± 0.07^a^	0.64 ± 0.04^b^	nd
p-Coumaric acid	0.76 ± 0.6^a^	0.60 ± 0.03^a^	1.07 ± 0.02^a^	1.91 ± 0.19^b^	1.44 ± 0.03^c^	2.49 ± 0.08^a^	0.06 ± 0.00^b^	0.08 ± 0.01^a^	nd
Caffeic acid	nd	0.24 ± 0.01	nd	nd	0.34 ± 0.01	nd	nd	0.08 ± 0.00	nd
Ferulic acid	14.55 ± 1.85^b^	12.34 ± 0.26^b^	20.21 ± 1.44^a^	0.59 ± 0.16^c^	13.03 ± 0.45^b^	21.31 ± 0.26^a^	nd	3.34 ± 0.16^b^	4.31 ± 0.17^a^
Sinapic acid isomers	nd	1.76 ± 0.05^a^	1.69 ± 0.12^a^	nd	1.17 ± 0.07	nd	nd	0.88 ± 0.06	nd

nd: not detected; hex: hexoside; pen: pentoside; dihex: dihexoside.

## Supplementary Materials

The following are available online, Table S1: Concentration of main free and bound PCs in the experimental cookies prepared using standard (*S. cerevisiae*) or lactic (*S. cerevisiae* and LAB) fermentation; Table S2: Statistical analysis of the concentration of main PCs in the <3 kDa digested fractions of the experimental cookies; Table S3: Summary of ANOVA results on the main PCs in the < 3 kDa digested fractions of the experimental cookies.
