# Supplementary material for: (Poly)phenolic Content and Profile and Antioxidant Capacity of Whole-Grain Cookies are Better Estimated by Simulated Digestion than Chemical Extraction
Source: Molecules. 2020 Jun 17;25(12):2792. doi: 10.3390/molecules25122792 (PMC7355583; doi:10.3390/molecules25122792)
Supplement: Supplementary file 1 [file molecules-25-02792-s001.pdf]

molecules

MDPI

SUPPLEMENTARY DATA

Table S1. Concentration of main free and bound PCs in the experimental cookies prepared using standard or sourdough fermentation. Values are expressed as mean values ± SD (n=4). Statistical analysis was by two-way ANOVA followed by Tukey's HSD test. Different letters in the same column indicate statistical significance ($p<0.05$).

	**Apigenin-*C*-hex-*C*-pen (1st)**	**Apigenin-*C*-hex-*C*-pen (2nd)**	***p*-Coumaric acid**	**Ferulic acid**	**Diferulic acid**	**Sinapic acid isomers #**
			**Free compounds (µg/g of cookie)**			
DUR_S	16.92 ± 0.65[b]	52.37 ± 1.64[b]	nd	nd	nd	nd
DUR_L	22.75 ± 2.89[a]	68.91 ± 1.98[a]	nd	24.16 ± 2.02[a]	nd	nd
KHO_S	8.07 ± 1.69[c,d]	28.15 ± 8.30[c]	nd	nd	nd	nd
KHO_L	10.00 ± 1.41[c]	33.47 ± 7.14[c]	2.68 ± 0.23	17.80 ± 3.81[b]	nd	nd
KAM_S	5.86 ± 1.71[d]	17.58 ± 1.01[d]	nd	nd	nd	nd
KAM_L	4.58 ± 0.28[d]	14.55 ± 0.41[d]	nd	nd	nd	nd
			**Bound compounds (µg/g of cookie)**			
DUR_S	nd	nd	6.90 ± 0.28[c]	551.27 ± 22.78[a]	577.70 ± 37.82[a,b]	20.26 ± 3.26[a]
DUR_L	nd	nd	7.26 ± 0.57[c]	558.88 ± 13.18[a]	589.48 ± 95.17[a,b]	22.32 ± 1.22[a]
KHO_S	nd	nd	13.18 ± 0.97[b]	491.97 ± 10.88[b]	529.13 ± 6.49[b,c]	9.49 ± 1.12[c]
KHO_L	nd	nd	15.12 ± 0.31[a]	538.10 ± 7.38[a]	655.78 ± 10.29[a]	14.82 ± 1.54[b]
KAM_S	nd	nd	7.73 ± 0.81[c]	326.70 ± 12.03[c]	372.27 ± 6.56[d]	11.22 ± 1.08[b,c]
KAM_L	nd	nd	7.10 ± 0.30[c]	338.31 ± 18.94[c]	432.96 ± 26.09[c,d]	11.37 ± 2.46[b,c]

nd: not detected; hex: hexoside; pen: pentoside; # sum of sinapic acid and its isomer.

Table S2. Statistical analysis of the concentration of main PCs in the < 3 KDa digested fractions of the experimental cookies. Values are expressed as mean ± SD (n=3) and are from Tables 2 and 3. Statistical analysis was by two-way ANOVA followed by Tukey's HSD test. Different letters in the same column indicate statistical significance ($p<0.05$).

**Compound**	**DUR$_S$ (μg/g of cookie)**	**DUR$_L$ (μg/g of cookie)**	**KHO$_S$ (μg/g of cookie)**	**KHO$_L$ (μg/g of cookie)**	**KAM$_S$ (μg/g of cookie)**	**KAM$_L$ (μg/g of cookie)**
Apigenin-*C*-hex-*C*-pen (1st)	30.43±2.29^{a}	31.47±1.32^{a}	17.73±0.66^{b}	17.21±0.23^{b}	10.70±0.36^{c}	10.77±0.29^{c}
Apigenin-*C*-hex-*C*-pen (2nd)	73.87±0.50^{b}	81.38±0.20^{a}	49.84±0.84^{c}	49.37±1.15^{c}	26.65±0.15^{d}	25.86±0.14^{d}
Apigenin-*C*-dihex (1st)	2.02±0.15^{a}	2.06±0.17^{a}	1.25±0.02^{b}	1.05±0.07^{b}	nd	nd
Apigenin-*C*-dihex (2nd)	2.38±0.02	nd	nd	nd	nd	nd
Apigenin-*C*-dihex (3rd)	nd	2.35±0.24	nd	nd	nd	nd
p-Hydroxybenzoic acid	nd	nd	nd	nd	nd	nd
p-Coumaric acid	0.64±0.06^{c}	1.07±0.02^{b}	1.07±0.15^{b}	2.49±0.08^{a}	0.41±0.02^{c}	nd
Caffeic acid	nd	nd	nd	nd	nd	nd
Ferulic acid	4.24±0.79^{b}	20.21±1.44^{a}	5.30±1.21^{b}	21.31±0.26^{a}	3.40±0.35^{b}	4.31±0.17^{b}
Sinapic acid isomers	nd	1.69±0.12	nd	nd	nd	nd

nd: not detected; hex: hexoside; pen: pentoside; dihex: dihexoside.

Table S3. Summary of ANOVA results on the main PCs in the < 3 KDa digested fractions of the experimental cookies.

Source	*df*	MS	*F*	p
**Apigenin-*C*-hexoside-*C*-pentoside (1st)**				
WG cereal	2	635.2	491.1	<0.0001
fermentation	1	0.2450	0.1894	0.6711
WG cereal × fermentation	2	0.9800	0.7577	0.4899
**Apigenin-*C*-hexoside-*C*-pentoside (2nd)**				
WG cereal	2	3967	9834	<0.0001
fermentation	1	19.85	49.20	<0.0001
WG cereal × fermentation	2	32.87	81.48	<0.0001
**Apigenin-*C*-dihexoside (1st)**				
WG cereal	2	6.276	418.4	<0.0001
fermentation	1	0.01280	0.8533	0.3738
WG cereal × fermentation	2	0.02480	1.653	0.2322
***p*-Coumaric acid**				
WG cereal	2	3.759	375.9	<0.0001
fermentation	1	1.037	103.7	<0.0001
WG cereal × fermentation	2	1.259	125.9	<0.0001
**Ferulic acid**				
WG cereal	2	160.3	222.2	<0.0001
fermentation	1	540.2	748.6	<0.0001
WG cereal × fermentation	2	113.6	157.3	<0.0001

df: degrees of freedom; MS = mean squares.
